# Editorial: The mechanism and interventions of aging-related cognitive impairment in perioperative context

**DOI:** 10.3389/fnagi.2023.1174890

**Published:** 2023-03-24

**Authors:** Shiyong Li, Dong-Xin Wang, Ping Zhao, Ailin Luo

**Affiliations:** ^1^Department of Anesthesiology, Hubei Key Laboratory of Geriatric Anesthesia and Perioperative Brain Health, and Wuhan Clinical Research Center for Geriatric Anesthesia, Tongji Hospital, Tongji Medical College, Huazhong University of Science and Technology, Wuhan, China; ^2^Department of Anesthesiology, Peking University First Hospital, Beijing, China; ^3^Department of Anesthesiology, Shengjing Hospital of China Medical University, Shenyang, China

**Keywords:** elderly patients, aging-related cognitive decline, postoperative delirium, postoperative cognitive dysfunction, neuroinflammation, anesthesia, gut microbiota

Age brings wisdom, but it also brings a growing challenge to health. As a result, a growing number of health problems need to be managed with invasive or mini-invasive procedures in order to relieve discomfort or restore functional status. It was estimated in 2015 that 313 million surgical procedures were performed worldwide each year (Meara et al., 2015). With the accelerated aging of the population, nearly 20% of patients subject to surgery are aged over 65 years old. In some countries, the proportions exceed 50%. For surgical individuals, advanced age is an independent predictor of unfavorable long-term outcomes. One concern for older patients is whether general anesthetics, which are pain-free and make patients lose consciousness, could lead to irreversible injury to the vulnerable brain. Two anesthesia-related postoperative neurological complications have been commonly identified: postoperative cognitive dysfunction (POCD) and postoperative delirium (POD). Accumulating findings suggest that advanced age, comorbidities, frailty, preoperative cognitive status, level of education, and other factors contribute to these two complications. Both prolonged POCD and POD are associated with prolonged hospital stay, reduced quality of life, and increased burden on caregivers and the medical system; thus, these problems have attracted growing attention. However, the underlying mechanisms are still being debated, and preventive measures need to be further explored. We highlight here three specific aims of the present topic: (1) to update the mechanisms underlying postoperative cognitive dysfunction with comorbidities such as type 2 diabetes, Alzheimer's disease, obstructive sleep apnea, and Parkinson's disease; (2) to explore the promising developments to prevent this neurological complication in clinical and preclinical research; (3) to gather studies that aim to develop behavior evaluation toolkits that are specific to the aged population with visual or auditory decline.

To date, neuroinflammation has perhaps been the least controversial neuropathological mechanism underlying postoperative neurocognitive disorders (PND). In previous studies, interleukin-1β, tumor necrosis factor-alpha, and interleukin-6 (IL-6) were found to be involved in PND experimental models (Cibelli et al., 2010; Terrando et al., 2010; Hu et al., 2018). Based on the series of studies investigating the role IL-6 in PND, Chang and Maze summarized preclinical and clinical data to explore the link between an increased level of IL-6 and PND. They identified that IL-6 trans-signaling in hippocampal CA1 neurons mediated surgery-induced memory impairment in adult male mice following tibial fracture under general anesthesia (Hu et al., 2022). These data suggest that olamkicept, a highly selective IL-6 trans-signaling blocker, could be repurposed to treat PND in the surgical population, considering that it is efficacious and safe in treating inflammatory bowel disease (Schreiber et al., 2021), in which IL-6 trans-signaling has been shown to mediate the pathological mechanism.

Regarding the effects of general anesthetics on the neurocognitive function, most of the available evidence comes from preclinical studies. In the clinical setting, it is difficult to differentiate the effect of anesthesia from surgical stress on the occurrence of postoperative cognitive abnormality (Li T. et al., 2021; Li Y. W. et al., 2021), although several intravenous general anesthetics are likely to be associated with less neurological symptoms. However, it is undeniable that general anesthetics change the conscious state of the recipient. Yang et al. found that propofol, when administered for seven consecutive days, reduced synaptic plasticity and cognitive dysfunction by upregulating FBXO22 in adult mice. Moreover, a previous study found that propofol impaired the processivity of the microtubule-based motor kinesin of neurons (Bensel et al., 2017). Although it is unclear whether the effect of propofol is associated with POD, propofol does change the cellular anatomical structure. In fact, we cannot rule out the possibility that propofol induces cognitive decline in the aged population, although some clinical findings suggest that patients receiving intravenous anesthesia have a lower incidence of POD.

Surgery for hip fracture, the so-called last fracture in the lifespan of human beings, is considered to be the high-risk surgical procedure of PND. From the perspective of PND incidence, there is no definite evidence that supports the selection of a specific anesthetic regimen. In the current topic, Tang et al. found that a fascia iliaca compartment block (FICB) mitigated postoperative cognitive impairment in older patients, accompanied by reduction in tau and Aβ in the peripheral blood. They partially attributed the decreased incidence of cognitive impairment to improved pain relief in the surgical patients. The gut microbiome plays essential roles in human health and disease (Minerbi and Shen, 2022). Emerging findings suggest it may also be associated with PND in preclinical studies. Bi et al. preoperatively screened six genera bacterium enrichments and found that the corresponding metabolites, such as acetic acid, arachidic acid, and pyrophosphate, were decreased in older patients following orthopedic surgery. These findings suggest that the changed composition of the gut microbiome and metabolites could be promising predictors of PND.

In clinical settings, screening the cognitive status is the prerequisite to taking prophylactic measurements. Until now, there has not been a feasible and brief screening tool. Huang et al. tried to analyze the preoperative information between patients postoperatively diagnosed as POCD and non-POCD patients and then establish a scoring system (nomogram) to predict POCD in older patients undergoing a gastrointestinal tumor resection. This screening tool may be imperfect, but it at least provides an insightful reference for studies in this field for the future. Preoperatively existing cognitive impairment is a high-risk factor of POCD in the elderly population. In the present topic, Mano et al. found that preoperative cognitive status was negatively correlated with the correct execution of perioperative instructions, which might increase the likelihood of postoperative complications. This study indicated that measures to guarantee the correct implementation of perioperative instructions might decrease the incidence of undesirable outcomes in patients with preexisting cognitive impairment.

In summary, the aim of the present Research Topic was to explore the potential mechanisms of perioperative cognitive impairment and the potential measures to prevent its occurrence in preclinical and clinical settings. These manuscripts provide important new insights on the topic. In the future, we hope that more studies will be performed to examine the association between preexisting comorbidities and postoperative cognitive impairment.

## Author contributions

SL, D-XW, PZ, and AL decided the layout, wrote the manuscript, and acted as guest editors for this Research Topic. All authors read and approved the final manuscript.
